# Prediction of new onset of end stage renal disease in Chinese patients with type 2 diabetes mellitus – a population-based retrospective cohort study

**DOI:** 10.1186/s12882-017-0671-x

**Published:** 2017-08-01

**Authors:** Eric Yuk Fai Wan, Daniel Yee Tak Fong, Colman Siu Cheung Fung, Esther Yee Tak Yu, Weng Yee Chin, Anca Ka Chun Chan, Cindy Lo Kuen Lam

**Affiliations:** 1 Department of Family Medicine and Primary Care, the University of Hong Kong, 3/F Ap Lei Chau Clinic, 161 Main Street, Ap Lei Chau, Hong Kong; 2 School of Nursing, the University of Hong Kong, Pok Fu Lam, Hong Kong

**Keywords:** Type 2 diabetes mellitus, Prediction, Risk, End stage renal disease, Primary care

## Abstract

**Background:**

Since diabetes mellitus (DM) is the leading cause of end stage renal disease (ESRD), this study aimed to develop a 5-year ESRD risk prediction model among Chinese patients with Type 2 DM (T2DM) in primary care.

**Methods:**

A retrospective cohort study was conducted on 149,333 Chinese adult T2DM primary care patients without ESRD in 2010. Using the derivation cohort over a median of 5 years follow-up, the gender-specific models including the interaction effect between predictors and age were derived using Cox regression with a forward stepwise approach. Harrell’s C-statistic and calibration plot were applied to the validation cohort to assess discrimination and calibration of the models.

**Results:**

Prediction models showed better discrimination with Harrell’s C-statistics of 0.866 (males) and 0.862 (females) and calibration power from the plots than other established models. The predictors included age, usages of anti-hypertensive drugs, anti-glucose drugs, and Hemogloblin A1c, blood pressure, urine albumin/creatinine ratio (ACR) and estimated glomerular filtration rate (eGFR). Specific predictors for male were smoking and presence of sight threatening diabetic retinopathy while additional predictors for female included longer duration of diabetes and quadratic effect of body mass index. Interaction factors with age showed a greater weighting of insulin and urine ACR in younger males, and eGFR in younger females.

**Conclusions:**

Our newly developed gender-specific models provide a more accurate 5-year ESRD risk predictions for Chinese diabetic primary care patients than other existing models. The models included several modifiable risk factors that clinicians can use to counsel patients, and to target at in the delivery of care to patients.

**Electronic supplementary material:**

The online version of this article (doi:10.1186/s12882-017-0671-x) contains supplementary material, which is available to authorized users.

## Background

Current prevalence of diabetes mellitus is one in 11 adults, affecting 415 million people, and it is estimated to increase to 642 million all over the world [1]. Diabetes is the leading cause of end-stage renal disease (ESRD), which is an irreversible loss of renal function and is fatal without receiving renal replacement therapy [2, 3]. The coexistence of diabetes and ESRD elevate the risk of mortality significantly [4–6]. Although the number of patients with ESRD account for only 0.1%–0.2% of the total population in developed countries, health spending on renal failure was 2–3% of the total healthcare expenditure [7]. Given available effective treatments in reduction of development and progression of diabetic kidney disease [8], identification of diabetic patients who are at high risk of ESRD is needed to allow target delivery of proper healthcare and facilitate service policy planning.

Several national guidelines including the National Institute for Health and Care Excellence from UK and the American Diabetes Association recommend the regular screening for diabetic kidney disease in diabetic patients [8, 9]. On one hand, some prediction models established from general population like QKidney model [10–12] included diabetes as one of the predictors only, without examining other clinical indicators such as haemoglobin A1c (HbA1c), estimated glomerular filtration rate (eGFR) and urine albumin to creatinine ratio (ACR), which are associated with the risk of developing ESRD [11, 13, 14]. Given the 3 to 5 folds higher risk of ESRD incidence in diabetic patients compared to non-diabetic patients [10], the type and magnitude of the association of the predictors for ESRD for diabetic population may differ from those for non-diabetic population. Only a few studies have established risk prediction models in ESRD such as the New Zealand Diabetes Cohort Study and the Action in Diabetes and Vascular Disease: Preterax and Diamicron-MR Controlled Evaluation (ADVANCE). These studies were done predominantly in a non-Chinese diabetic population [4, 15]. Studies have shown different ESRD incidence rates in different racial groups including Chinese populations [16–18]. Hence, these models may not be applied to a Chinese diabetic population. While a ESRD risk prediction model was derived from Chinese diabetic patients managed in secondary care, one of the predictors in this model, haematocrit, is not routinely available in primary care and thus this model may be more suitable to be applied in secondary care than in primary care [19]. In addition, a previous study reported that there was a potential difference in predictors of ESRD incidence between male and female [10], but most of established models without stratifying gender may not obtain accurate predicted ESRD risk. Therefore, there is a need to develop a more accurate prediction model by stratifying gender for ESRD risk based on Chinese primary care patient with diabetes.

Since there were no population-based studies on ESRD risk prediction models specific to Chinese primary care diabetic population, the aim of this study was to develop a 5-year ESRD risk prediction model among Chinese patients with Type 2 DM (T2DM) in primary care.

## Methods

### Study design

This is a population-based retrospective cohort study. Subject inclusion criteria included (1) Chinese, (2) age between 18 and 79 years old, (3) clinically diagnosed with T2DM, and (4) no previous record of CVD and ESRD. All subjects received primary care services from one of the 74 general outpatient clinics of the Hong Kong Hospital Authority (HA) between 1 January 2010 and 31 December 2010 and their clinical data were retrieved from the administrative database of the HA. The HA is the largest governmental organisation managing at least half of DM patients under primary care in Hong Kong. Data were available from a territory-wide study for the evaluation of local diabetic programmes [20]. Diagnosis of T2DM was clinically identified by the International Classification of Primary Care-2 (ICPC-2) code of ‘T90’. ESRD was identified by the International Classification of Diseases, Ninth Edition, Clinical Modification (ICD-9-CM) of 250.3x, 585.x and 586.x, or eGFR <15 ml/min/1.73m^2^. Baseline was defined as the patient’s first attendance date of general outpatient clinics during January 2010 and 31 December 2010. Each patient was follow-up until the date of diagnosis of ESRD event, death or last follow-up as censoring in 30 November 2015, whichever occurred first.

### Potential predictors

The potential predictors included socio-demographics, disease characteristics, treatment modalities and clinical parameters. Socio-demographics included gender, age and smoking status. Disease characteristics consisted of self-reported duration of DM, diagnosed hypertension and presence of sight threatening diabetic retinopathy (STDR). Diagnosis of hypertension was identified by the ICPC-2 of K86 to K87. STDR included pre-proliferative, proliferative diabetic retinopathy or maculopathy. Treatment modalities consisted of the usages of anti-hypertensive drugs, anti-glucose oral drugs, insulin and lipid-lowering agents. Clinical parameters were body mass index (BMI), waist circumference, HbA1c, systolic blood pressure (SBP), diastolic blood pressure (DBP), lipid profile (low-density lipoprotein-cholesterol (LDL-C) and total cholesterol to high-density lipoprotein-cholesterol ratio (TC/ HDL-C ratio)), triglyceride, urine ACR and eGFR. All laboratory assays were performed in accredited laboratories by the College of American Pathologists, the Hong Kong Accreditation Service or the National Association of Testing Authorities, Australia.

### Data analysis

Missing data were handled by multiple imputation [21]. Specifically, each missing value was imputed five times using chained equation method. The same analysis was performed for each imputed dataset and the resulting five sets of results were aggregated by the Rubin’s rule [22].

The risk prediction models were developed separately for each gender since several studies discovered that there was a potential difference in predictors of ESRD incidence between male and female [23–28]. For each gender, all subjects were randomly divided on a 2:1 basis, with two-third subjects being the derivation cohort to develop the risk prediction models, and the remaining one-third being the validation cohort to validate the derived prediction models. Independent t-tests or chi-square tests were used to evaluate if there was any significant difference in potential predictors between the two cohorts.

Cox proportional hazards regression with forward stepwise method was performed to obtain a risk prediction model using the derivation cohort. The cutoff *p*-values for variable entering and leaving the model for each step were 0.05 and 0.1, respectively. Since previous literature showed that some predictors such as blood pressure had curvilinear relationship with adverse event [29, 30], the quadratic terms of these predictors were also considered in our model. Furthermore, another study revealed that the effects of some factors, such as blood pressure, may vary across age [29]. Thus, the interaction effects between age and the predictors were also assessed. The proportional hazards assumption was checked by examining plots of the scaled Schoenfeld residuals against time for the predictors. Any non-random pattern implies a violation of the proportional hazards assumptions and thereafter transformation of covariates would be attempted. All models in our study fulfilled proportional hazards assumption.

The performance of our model, ADVANCE and New Zealand ESRD risk scores for T2DM was compared using the validation cohorts [4, 15]. The New Zealand model were developed based on European, Maori, Pacific, East Asian, Indo-Asian from New Zealand and included 10 predictors which were age, gender, duration of DM, smoking status, HbA1c, SBP, urine ACR, eGFR, history of CVD and ethnicity. Meanwhile, the ADVANCE model were established across 20 countries and included 7 predictors which were gender, HbA1c, SBP, urine ACR, eGFR, retinopathy and education level. Since we did not have educational data, we assumed no attendance at education until at least 16 years of age in the ADVANCE risk model. For each model, the Harrell’s C statistic, D statistic and R^2^ statistic were computed to assess the predictive power. A Harrell’s C statistics of less than 0.7 indicates limited discriminating power, 0.7 to 0.9 is acceptable, and higher than 0.9 suggests strong discrimination of the predictive models [31]. The D statistic is a measure of discrimination with higher value implies better discrimination. The R^2^ statistic measures the explained variation in the model with higher value indicating better performance. After bootstrapping of size 500, the corresponding 95% confidence intervals (CIs) were obtained. The calibration plots were also displayed to compare the mean of predicted risk at 5 years with the observed ESRD risk, which was obtained by 5-year Kaplan-Meier estimate, by deciles of predicted risk.

All significance tests were two-tailed and those with *p*-values less than 0.05 were considered statistically significant. The statistical analysis was performed in STATA Version 13.0.

## Results

There were a total of 149,333 Chinese T2DM patients aged between 18 and 79 years receiving care in primary care clinics of HA between 1 January 2010 and 31 December 2010. After excluding 10,789 patients had CVD at baseline, 21,426 patients had ESRD at baseline and 609 patients had no follow-up record, the remaining 116,509 diabetic patients, including 54,472 males and 62,037 females were included in the main analysis. The three lowest data completion rates for STDR, urine ACR and waist circumference were 70%, 72% and 83%, respectively, while other potential predictors were higher than 90%. During a median follow-up period of 5 years (range: 0.04 to 6.04 years), the incidence rate of ESRD per 1000 person-years was 0.41 (95% CI: 0.40–0.43), while that in male and female groups were 0.50 (95% CI: 0.47–0.52) and 0.34 (95% CI: 0.32–0.36), respectively. For each gender, Table 1 compares the baseline characteristics between derivation and validation cohorts after multiple imputation. For the derivation cohorts, the mean ages for male and female were 61.4 and 62.7 years, respectively. The two cohorts did not show any significant differences in all potential predictors for each gender.

**Table 1:** Baseline characteristics of derivation and validation cohorts in male and female groups

Characteristics	Male		Female	
	Derivation cohort (*n* = 36,289)	Validation cohort (*n* = 18,145)	Derivation cohort (*n* = 41,347)	Validation cohort (*n* = 20,674)
Socio-demographics				
Age,years	61.4 ± 10.0	61.4 ± 10.0	62.7 ± 9.9	62.8 ± 9.8
Smoker	7627 (21.0%)	3843 (21.2%)	923 (2.2%)	411 (2.0%)
Disease characteristics				
Duration of T2DM, years	6.8 ± 6.1	6.9 ± 6.4	7.4 ± 6.7	7.4 ± 6.6
Diagnosed Hypertension	24,862 (68.5%)	12,488 (68.8%)	30,857 (74.6%)	15,436 (74.7%)
STDR	2111 (5.8%)	1057 (5.8%)	1961 (4.7%)	934 (4.5%)
Treatment modalities				
Anti-hypertensive drugs used	27,294 (75.2%)	13,573 (74.8%)	31,845 (77.0%)	15,980 (77.3%)
Anti-glucose oral drugs used	32,296 (89.0%)	16,102 (88.7%)	35,875 (86.8%)	17,977 (87.0%)
Insulin used	929 (2.6%)	475 (2.6%)	983 (2.4%)	509 (2.5%)
Lipid-lowering agents used	9016 (24.8%)	4639 (25.6%)	12,480 (30.2%)	6245 (30.2%)
Clinical parameters				
BMI, kg/m^2^	25.4 ± 3.8	25.5 ± 3.8	25.6 ± 4.2	25.5 ± 4.3
Waist circumference, cm	90.7 ± 19.8	90.8 ± 17.8	87.5 ± 20.8	87.6 ± 24.5
HbA1c, %	7.2 ± 1.3	7.2 ± 1.3	7.2 ± 1.2	7.2 ± 1.2
HbA1c, mmol/mol	55 ± 14.2	55 ± 14.2	55 ± 13.1	55 ± 13.1
SBP, mmHg	134.0 ± 15.9	134.2 ± 16.1	134.6 ± 16.5	134.7 ± 16.4
DBP, mmHg	77.0 ± 9.8	77.1 ± 9.7	73.7 ± 9.7	73.7 ± 9.6
LDL-C, mmol/L	2.9 ± 0.8	2.9 ± 0.8	3.0 ± 0.8	3.0 ± 0.8
TC/HDL-C ratio	4.3 ± 1.2	4.3 ± 1.2	4.0 ± 1.1	4.0 ± 1.1
Triglyceride, mmol/L	1.6 ± 0.9	1.6 ± 0.9	1.6 ± 0.9	1.6 ± 0.9
Urine ACR, mg/mmol	6.8 ± 40.6	7.0 ± 41.7	6.8 ± 37.9	7.2 ± 52.0
eGFR, ml/min/1.73m^2^				
≥ 90	26,683 (73.5%)	13,277 (73.2%)	29,839 (72.2%)	14,857 (71.9%)
60–89	8694 (24.0%)	4381 (24.1%)	9906 (24.0%)	5041 (24.4%)
< 60	912 (2.5%)	487 (2.7%)	1601 (3.9%)	776 (3.8%)

*T2DM* Type 2 Diabetes Mellitus, *STDR* Sight Threatening Diabetic Retinopathy, *BMI* Body Mass Index, *HbA1c* Haemogloblin A1c, *SBP* Systolic Blood Pressure, *DBP* Diastolic Blood Pressure, *LDL-C* Low Density Lipoprotein-Cholesterol, *TC* Total Cholesterol, *HDL-C* High-density Lipoprotein-Cholesterol, *ACR* Albumin/Creatinine Ratio, *eGFR* estimated Glomerular Filtration Rate

After selecting the predictors by forward stepwise method (shown in Additional file 1: Table S1a and 1b), Table 2 and Additional file 2: Table S2 show the estimated risk prediction models for each gender by Cox proportional hazards regression. Common predictors to both male and female included older age, usages of anti-hypertensive drugs, anti-glucose oral drugs and insulin, and increased SBP, urine ACR and eGFR. Moreover, quadratic effects of HbA1c and DBP were significantly associated with increased risk of ESRD. For male T2DM subjects, additional predictors of ESRD risk were smoking and presence of STDR. The magnitudes of the association for insulin and urine ACR also decreased across age. For female T2DM subjects, additional predictors included longer duration of T2DM and the quadratic effect of BMI. Similarly, the magnitude of the association for eGFR also decreased across age. To summarise, the developed risk prediction model for male included 11 predictors: age, smoking status, presence of STDR, usages of anti-hypertensive drugs, anti-glucose oral drugs and insulin, HbA1c, SBP, DBP, urine ACR and eGFR, whereas the one for female included 12 predictors: age, duration of T2DM, usages of anti-hypertensive drugs, anti-glucose oral drugs and insulin, BMI, HbA1c, SBP, DBP, urine ACR and eGFR. The formulae for the derivation of the predicted ESRD risk were shown in Additional file 3: Table S3.

**Table 2:** Cox regression models for predictors of developing end stage renal disease in derivation cohort

Predictors	Male			Female		
	HR^a^	95%CI	*P*-value	HR^a^	95%CI	*P*-value
Socio-demographics						
Age, years	1.06	(1.05,1.08)	<0.001*	1.03	(1.02,1.05)	<0.001*
Smoker (Non-smoker)	1.29	(1.11,1.50)	0.001*			
Disease characteristics						
Duration of T2DM, years				1.01	(1.00,1.02)	0.046*
STDR (No)	1.47	(1.18,1.84)	0.001*			
Treatment modalities						
Anti-hypertensive drugs used (No)	1.54	(1.21,1.97)	0.001*	1.66	(1.21,2.26)	0.002*
Oral drug (No)	1.37	(1.07,1.75)	0.011*	1.98	(1.43,2.75)	<0.001*
Insulin drug (No)	9.79	(1.66,57.62)	0.012*	1.73	(1.30,2.29)	<0.001*
Clinical parameters						
BMI, kg/m^2^				0.85	(0.77,0.95)	0.002*
BMI^2^, kg/m^2^				1.003	(1.001,1.005)	0.001*
HbA1c, %	0.79	(0.64,0.98)	0.036*	0.70	(0.49,0.99)	0.046*
HbA1c^2^, %	1.02	(1.01,1.03)	0.002*	1.03	(1.01,1.05)	0.013*
SBP, mmHg	1.01	(1.01,1.02)	<0.001*	1.01	(1.00,1.01)	0.025*
DBP, mmHg	0.93	(0.88,0.98)	0.009*	0.92	(0.86,0.98)	0.007*
DBP^2^, mmHg	1.0004	(1.0000,1.0008)	0.039*	1.001	(1.000,1.001)	0.017*
ln(Urine ACR + 1), mg/mmol	3.15	(2.11,4.69)	<0.001*	1.45	(1.37,1.53)	<0.001*
eGFR (>90 ml/min/1.73m^2^)						
60-89 ml/min/1.73m^2^	2.45	(2.08,2.88)	<0.001*	2.00	(0.38,10.51)	0.412
< 60 ml/min/1.73m^2^	8.76	(7.08,10.84)	<0.001*	112.06	(23.97,523.76)	<0.001*
Age interaction term						
Age*eGFR(>90 ml/min/1.73m^2^)						
60-89 ml/min/1.73m^2^				1.003	(0.979,1.028)	0.816
< 60 ml/min/1.73m^2^				0.97	(0.95,0.99)	0.006*
Age*insulin	0.97	(0.95,1.00)	0.028*			
Age* ln(Urine ACR + 1)	0.99	(0.98,0.99)	<0.001*			

*T2DM* Type 2 Diabetes Mellitus, *STDR* Sight Threatening Diabetic Retinopathy, *BMI* Body Mass Index, *HbA1c* Hemogloblin A1c, *SBP* Systolic Blood Pressure, *DBP* Diastolic Blood Pressure, *TC* Total Cholesterol, *HDL-C* High-density Lipoprotein-Cholesterol, *ACR* Albumin/Creatinine Ratio, *eGFR* estimated Glomerular Filtration Rate, *T2DM* Type 2 Diabetes Mellitus, *HR* Hazard Ratio

* Significant difference (*P* < 0.05)

^a^ HR > 1 indicates greater risk of event occurrence

**Fig. 1:**
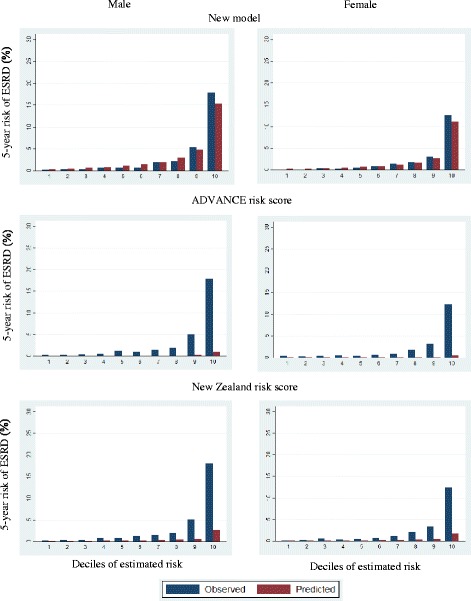
Calibration plots for observed and predicted 5-year risks of end stage renal disease (ESRD)

Table 3 compares the performance of the newly developed prediction models with ADVANCE and New Zealand ESRD risk scores using validation cohort. In terms of prediction power, both the new and the New Zealand models performed better than the ADVANCE model for female while there was no significant difference among the three models for male. Figure 1 displays the calibration plot on the predicted risk and the observed ESRD risk at 5 years in each gender. The new model demonstrated better calibration than the other models.

**Table 3:** Performance of new and existing end stage renal disease risk models in validation cohort for predicting 5-year risk of end stage renal disease

Validation statistics	New model	ADVANCE model	New Zealand model
Male			
Harrell’s C statistic	0.866 (0.849,0.882)	0.858 (0.840,0.876)	0.863 (0.846,0.880)
Difference in Harrell’s C statistic in comparison with new model	-	0.008	0.002
D statistic	2.458 (2.298,2.595)	2.429 (2.270,2.577)	2.482 (2.337,2.615)
R^2^	59.1 (55.9,61.7)	58.5 (55.1,61.6)	59.5 (56.6,62.2)
Female			
Harrell’s C statistic	0.862 (0.845,0.880)	0.844 (0.822,0.865)	0.861 (0.842,0.880)
Difference in Harrell’s C statistic in comparison with new model	-	0.019*	0.001
D statistic	2.410 (2.228,2.563)	2.366 (2.201,2.531)	2.493 (2.317,2.655)
R^2^	58.1 (54.4,61.2)	57.2 (53.1,61.2)	59.7 (56.1,63.1)

The brackets represented 95% confidence interval of corresponding validation statistic

* Significant difference in Harrell’s C statistic (*P*-value <0.05)

## Discussion

This is the first study to develop the prediction model for ESRD risk among Chinese population-based primary care patient with diabetes. Our findings showed that current ESRD risk prediction models such as ADVANCE and New Zealand had poor calibration power, which substantially underestimated the actual risk of ESRD in Chinese diabetic population. Our results showed that the prediction model for ESRD should be ethnic-specific and gender-specific. Moreover, we confirmed the importance of eGFR and urine ACR in predicting ESRD and found that the renal impairments interacted with age on the risk of ESRD for Chinese diabetic patients. Our model could identify diabetic patients who are at high risk of ESRD in order to counsel patients, allow target delivery of proper healthcare and facilitate service policy planning.

In comparison to our model, the ADVANCE and New Zealand models had comparable discriminatory power but lack of calibration power, indicating the apparent discrepancy between observed and predicted risks. As a consequence, the ultimate goal of the earlier identification for patients at high risk of ESRD may be defeated by the inaccurate predicted risk. Current results demonstrated that the ADVANCE and New Zealand models underestimated risk among Chinese diabetic population. Ethnic disparities in the risk of ESRD may be a potential explanation for this discrepancy. Although the ADVANCE model showed no difference in the risk of ESRD between Asian and non-Asian, numerous multi-ethnic studies showed that Asian diabetic patients had doubled the risk of albuminuria compared to Caucasian diabetic patients [18, 32, 33]. Meanwhile, the New Zealand model included ethnicity as a predictor to allow the modification for the various risks of ESRD in different ethnic groups. Nevertheless, only a small proportion of East Asian subjects (717 out of 25,736) was included in their sample and it may not be fully representative of a Chinese diabetic population. Moreover, a multinational study conducted by the World Health Organisation found that Chinese patients had a higher prevalence of proteinuria compared to other populations [34, 35]. A recent epidemiological study also illustrated that the risk of ESRD in Asian diabetic population varied widely between Chinese, Malay and Asian Indian [36]. The ethnic differences in disease profile may be attributable to genetic variants and the environmental factors such as health care policy and cultural behaviours between diabetic populations [34–37], and thus the prediction model for ESRD should be ethnic-specific. The external validation should be conducted in the future study in order to validate our model in other Chinese populations.

The key novelty of our study was to extend on the previous models for the risk of ESRD by stratifying gender and including age effect on the renal impairment measured by urine ACR and eGFR. This study supports prior observations that higher risk of renal impairment in male compared to female [23–28] and the current results prolonged this manifestation that the predictors for the risk of ESRD between genders were different. The reasons may be related to the genetic diversity between genders in kidney structure and function, receptor mediated influence of sex steroids on glomerular structure, as well as response on the synthesis and discharge of cytokines and growth factors [26, 27, 38]. The different modifiable risk factors for male and female may also suggest different approaches and therapies for the prevention of ESRD. Meanwhile, a meta-analysis involving more than 1.5 million patients in different countries from 45 cohorts conducted by the Kidney Disease, Improving Global Outcomes (KDIGO) showed that urine ACR and eGFR were independent risk factors for both progression of chronic kidney disease and ESRD [39]. Through the stringent evaluation with an almost inexhaustible list of clinical variables, our finding confirmed that the effects of urine ACR and eGFR were not diminished by other variables. In addition, the results of the present study illustrated the interaction effect between age and renal impairment on the incidence of ESRD, which indicated that the impacts of urinary albumin and kidney function were diluted by older age. This phenomenon may be understandable because the severity of renal impairment in elderly may be certainly high, and thus there is a relatively small room to decline as a result of lower changes in urine ACR and eGFR among elder patients compared to younger patients. An aforementioned observational study conducted in the United States also displayed that there is a continuous trend with a slow progressive eGFR decline observed among the general population after age of 40 [40]. Other predictors in our model were well discussed in the literatures [26, 27, 41]. A further study should be warranted to confirm the interaction effect on the incidence of ESRD between age and renal impairments, and the curvilinear relationship between risk of ESRD and the predictors including HbA1c and BP.

### Strengths and limitations of this study

There were several strengths in the current study. Firstly, using a large primary care Chinese T2DM population in current study is highly representative of the Hong Kong Chinese diabetic population managed in primary care setting. Secondly, the clinical and laboratory data were systemically extracted from the HA’s computerised administrative database, which were more reliable and accurate. Finally, multiple imputations were conducted to substitute the missing data so as to capture less biased results.

On the other hand, this study has several limitations. Firstly, retrospective rather than prospective was conducted in our study design, which may cause some bias to the results. Secondly, the risk of ESRD may be different between non-smokers and past smokers, but our non-smokers including past smokers was potentially bias to the results. Thirdly, only 5-year ESRD predicted risk was available in our model. A future study with longer follow-up periods of 10-year is necessary to develop the model that can forecast longer-term ESRD risks for diabetic populations. Fourthly, the developed models consisted of 11–12 predictors, which may be difficult to be applied in clinical practices. Lastly, only internal validation but not external validation was available in the current study. An external validation should be warranted to validate our model by using Chinese population in other regions.

## Conclusions

Our newly developed gender-specific models provide a more accurate and valid 5-year ESRD risk predictions for Chinese diabetic primary care patients than other currently existing models. We confirmed the importance of eGFR and urine ACR in predicting ESRD and found the renal impairments interacted with age on the risk of ESRD for Chinese diabetic patients. Our model could identify diabetic patients who are at high risk of ESRD and included several modifiable risk factors such as smoking and blood pressure in order to counsel patients, allow target delivery of proper healthcare and facilitate service policy planning.

## Additional files

Additional file 1: Table S1a. Summary of Cox proportional hazards regression with forward stepwise method for male. b. Summary of Cox proportional hazards regression with forward stepwise method for female. (DOCX 19 kb)

Additional file 2: Regression coefficients of developing end stage renal disease in derivation cohort. (DOCX 17 kb)

Additional file 3: Formulae for the estimation of 5-year risk of end stage renal disease. (DOCX 14 kb)

## Abbreviations

ACR: Albumin/Creatinine Ratio
ADVANCE: Action in Diabetes and Vascular Disease: Preterax and Diamicron-MR Controlled Evaluation
BMI: Body Mass index
CHD: Coronary heart disease
CI: Confidence interval
CVD: Cardiovascular diseases
DBP: Diastolic blood pressure
DM: Diabetes mellitus
eGFR: Estimated glomerular filtration rate
ESRD: End stage renal disease
HA: Hospital authority
HbA1c: Haemoglobin A1c
HR: Hazard ratio
ICD-9-CM: International classification of diseases, ninth edition, clinical modification
ICPC-2: International classification of primary care-2
LDL-C: Low density lipoprotein-cholesterol
SBP: Systolic blood pressure
SD: Standard deviation
STDR: Sight threatening diabetic retinopathy
T2DM: Type 2 diabetes mellitus
TC/HDL-C ratio: Total cholesterol to high density lipoprotein-cholesterol ratio
