# Supplementary material for: Prediction of new onset of end stage renal disease in Chinese patients with type 2 diabetes mellitus – a population-based retrospective cohort study
Source: BMC Nephrol. 2017 Aug 1;18:257. doi: 10.1186/s12882-017-0671-x (PMC5539616; doi:10.1186/s12882-017-0671-x)
Supplement: Supplementary file 1 — Summary of Cox proportional hazards regression with forward stepwise method for male. b. Summary of Cox proportional hazards regression with forward stepwise method for female. (DOCX 19 kb) [file 12882_2017_671_MOESM1_ESM.docx]

Supplementary Table 1a. Summary of Cox proportional hazards regression with forward stepwise method for male

| Step | Male | | | | |
| --- | --- | --- | --- | --- | --- |
|  | Variable entered | Hazard ratio | p-value | AIC | C-statistic |
| 1 | ln(Urine ACR+1) | 1.916 | <0.001 | 31925.8 | 0.7472 |
| 2 | Age | 1.068 | <0.001 | 31361.6 | 0.7883 |
| 3 | eGFR | (60-89) 2.555  (<60) 9.196 | <0.001 | 30573.4 | 0.8221 |
| 4 | HbA1c | 1.141 | <0.001 | 30520.1 | 0.8260 |
| 5 | HbA1c^2^ | 1.018 | 0.002 | 30513.1 | 0.8273 |
| 6 | Anti-hypertensive drugs used | 1.695 | <0.001 | 30496.2 | 0.8289 |
| 7 | STDR | 1.598 | <0.001 | 30472.7 | 0.8314 |
| 8 | Smoker | 1.304 | <0.001 | 30466.3 | 0.8319 |
| 9 | SBP | 1.005 | 0.008 | 30459.9 | 0.8332 |
| 10 | DBP | 0.983 | <0.001 | 30452.6 | 0.8342 |
| 11 | DBP^2^ | 1.0004 | 0.030 | 30457.1 | 0.8343 |
| 12 | Anti-glucose oral drugs used | 1.358 | 0.014 | 30461.2 | 0.8353 |
| 13 | Insulin used | 1.367 | 0.030 | 30465.9 | 0.8364 |
| 14 | Age* ln(Urine ACR+1) | 0.988 | <0.001 | 30426.8 | 0.8363 |
| 15 | Age*insulin | 0.971 | 0.028 | 30428.7 | 0.8374 |

T2DM = Type 2 Diabetes Mellitus; STDR = Sight Threatening Diabetic Retinopathy; HbA1c = Hemoglobin A1c; SBP = Systolic Blood Pressure; DBP = Diastolic Blood Pressure; ACR = Albumin/Creatinine Ratio; eGFR = estimated Glomerular Filtration Rate;; AIC = Akaike Information Criterion

Supplementary Table 1b. Summary of Cox proportional hazards regression with forward stepwise method for female

| Step | Female | | | | |
| --- | --- | --- | --- | --- | --- |
|  | Variable entered | Hazard ratio | p-value | AIC | C-statistic |
| 1 | eGFR | (60-89) 4.021  (<60) 31.26 | <0.001 | 24777.8 | 0.7843 |
| 2 | ln(Urine ACR+1) | 1.542 | <0.001 | 24325.5 | 0.8295 |
| 3 | Age | 1.032 | <0.001 | 24241.0 | 0.8345 |
| 4 | Insulin used | 2.100 | <0.001 | 24208.3 | 0.8370 |
| 5 | Anti-glucose oral drugs used | 2.147 | <0.001 | 24175.9 | 0.8388 |
| 6 | Anti-hypertensive drugs used | 1.727 | <0.001 | 24166.9 | 0.8401 |
| 7 | HbA1c | 0.736 | <0.001 | 24162.3 | 0.8423 |
| 8 | HbA1c^2^ | 1.024 | <0.001 | 24163.6 | 0.8431 |
| 9 | BMI | 0.838 | <0.001 | 24162.4 | 0.8432 |
| 10 | BMI^2^ | 1.003 | <0.001 | 24160.4 | 0.8450 |
| 11 | DBP | 0.923 | <0.001 | 24159.1 | 0.8449 |
| 12 | DBP^2^ | 1.00005 | 0.019 | 24158.3 | 0.8454 |
| 13 | SBP | 1.006 | 0.022 | 24157.8 | 0.8459 |
| 14 | Duration of T2DM | 1.012 | 0.043 | 24159.7 | 0.8460 |
| 15 | Age*eGFR | (60-89) 1.003  (<60) 0.969 | <0.001 | 24156.5 | 0.8465 |

T2DM = Type 2 Diabetes Mellitus; BMI = Body Mass Index; HbA1c = Hemoglobin A1c; SBP = Systolic Blood Pressure; DBP = Diastolic Blood Pressure; ACR = Albumin/Creatinine Ratio; eGFR = estimated Glomerular Filtration Rate; AIC = Akaike Information Criterion
