# Supplementary material for: Prediction of new onset of end stage renal disease in Chinese patients with type 2 diabetes mellitus – a population-based retrospective cohort study
Source: BMC Nephrol. 2017 Aug 1;18:257. doi: 10.1186/s12882-017-0671-x (PMC5539616; doi:10.1186/s12882-017-0671-x)
Supplement: Supplementary file 2 — Regression coefficients of developing end stage renal disease in derivation cohort. (DOCX 17 kb) [file 12882_2017_671_MOESM2_ESM.docx]

Supplementary Table 2. Regression coefficients of developing end stage renal disease in derivation cohort

| Predictors | Male | | | Female | | | |
| --- | --- | --- | --- | --- | --- | --- | --- |
|  | Coeff. | 95%CI | P-value | Coeff. | 95%CI | P-value |  |
| **Socio-demographics** |  |  |  |  |  |  |  |
| Age, years | 0.06 | (0.05,0.07) | <0.001* | 0.03 | (0.02,0.05) | <0.001* |  |
| Smoker (Non-smoker) | 0.26 | (0.11,0.41) | 0.001* |  |  |  |  |
|  |  |  |  |  |  |  |  |
| **Disease characteristics** |  |  |  |  |  |  |  |
| Duration of T2DM, years |  |  |  | 0.01 | (0.00,0.02) | 0.046* |  |
| STDR (No) | 0.39 | (0.16,0.61) | 0.001* |  |  |  |  |
|  |  |  |  |  |  |  |  |
| **Treatment modalities** |  |  |  |  |  |  |  |
| Anti-hypertensive drugs used (No) | 0.43 | (0.19,0.68) | 0.001* | 0.50 | (0.19,0.82) | 0.002* |  |
| Oral drug (No) | 0.32 | (0.07,0.56) | 0.011* | 0.68 | (0.36,1.01) | <0.001* |  |
| Insulin drug (No) | 2.28 | (0.51,4.05) | 0.012* | 0.55 | (0.26,0.83) | <0.001* |  |
|  |  |  |  |  |  |  |  |
| **Clinical parameters** |  |  |  |  |  |  |  |
| BMI, kg/m^2^ |  |  |  | -0.16 | (-0.26,-0.06) | 0.002* |  |
| BMI^2^, kg/m^2^ |  |  |  | 0.003 | (0.001,0.005) | 0.001* |  |
| HbA1c, % | -0.23 | (-0.45,-0.02) | 0.036* | -0.36 | (-0.71,-0.01) | 0.046* |  |
| HbA1c^2^, % | 0.02 | (0.01,0.03) | 0.002* | 0.03 | (0.01,0.05) | 0.013* |  |
| SBP, mmHg | 0.01 | (0.01,0.02) | <0.001* | 0.01 | (0.00,0.01) | 0.025* |  |
| DBP, mmHg | -0.07 | (-0.13,-0.02) | 0.009* | -0.09 | (-0.15,-0.02) | 0.007* |  |
| DBP^2^, mmHg | 0.0004 | (0.0000,0.0007) | 0.039* | 0.001 | (0.000,0.001) | 0.017* |  |
| ln(Urine ACR+1), mg/mmol | 1.15 | (0.75,1.55) | <0.001* | 0.37 | (0.32,0.42) | <0.001* |  |
| eGFR (>90ml/min/1.73m^2^) |  |  |  |  |  |  |  |
| 60-89ml/min/1.73m^2^ | 0.89 | (0.73,1.06) | <0.001* | 0.69 | (-0.97,2.35) | 0.412 |  |
| <60ml/min/1.73m^2^ | 2.17 | (1.96,2.38) | <0.001* | 4.72 | (3.18,6.26) | <0.001* |  |
|  |  |  |  |  |  |  |  |
| Age interaction term |  |  |  |  |  |  |  |
| Age*eGFR(>90ml/min/1.73m^2^) |  |  |  |  |  |  |  |
| 60-89ml/min/1.73m^2^ |  |  |  | 0.003 | (-0.021,0.027) | 0.816 |  |
| <60ml/min/1.73m^2^ |  |  |  | -0.03 | (-0.05,-0.01) | 0.006* |  |
| Age*insulin | -0.03 | (-0.06,0.00) | 0.028* |  |  |  |  |
| Age* ln(Urine ACR+1) | -0.01 | (-0.02,-0.01) | <0.001* |  |  |  |  |

T2DM = Type 2 Diabetes Mellitus; STDR = Sight Threatening Diabetic Retinopathy; BMI = Body Mass Index; HbA1c = Hemogloblin A1c; SBP = Systolic Blood Pressure; DBP = Diastolic Blood Pressure; TC = Total Cholesterol; HDL-C = High-density Lipoprotein-Cholesterol; ACR = Albumin/Creatinine Ratio; eGFR = estimated Glomerular Filtration Rate; T2DM = Type 2 Diabetes Mellitus; Coef. = Coefficient

* Significant difference (P < 0.05)
