# Supplementary material for: Prediction of new onset of end stage renal disease in Chinese patients with type 2 diabetes mellitus – a population-based retrospective cohort study
Source: BMC Nephrol. 2017 Aug 1;18:257. doi: 10.1186/s12882-017-0671-x (PMC5539616; doi:10.1186/s12882-017-0671-x)
Supplement: Supplementary file 3 — Formulae for the estimation of 5-year risk of end stage renal disease. (DOCX 14 kb) [file 12882_2017_671_MOESM3_ESM.docx]

Supplementary Table 3. Formulae for the estimation of 5-year risk of end stage renal disease

| Male | (1 – 0.9859326^exp[1.147257 × ln(Urine ACR + 1) + 0.0602995 × age + 0.98942913 × (eGFR≥60 and eGFR<90) + 2.169969 × (eGFR<60) – 0.2335348 × HbA1c + 0.0184585 × HbA1c2 + 0.4336443 × anti-hypertensive drugs used + 0.38614 × STDR + 0.2581414 × smoker + 0.0104007 × SBP – 0.0743492 × DBP + 0.0003842 × DBP2 + 0.3151005 × anti-glucose oral drugs used +2.281594 × insulin – 0.0109056 × age × ln(urine ACR + 1) – 0.0296208 × age × insulin used^) × 100% |
| --- | --- |
| Female | (1 – 0.9911678^exp[0.6931021 × (eGFR≥60 and eGFR<90) + 4.719013 × (eGFR<60) + 0.3696606 × ln(Urine ACR + 1) + 0.0342951 × age + 0.5458294 × insulin used + 0.6842409 × anti-glucose oral drugs used + 0.5039446 × anti-hypertensive drugs used – 0.3566864 × HbA1c + 0.0254595 × HbA1c2 – 0.1578451 × BMI + 0.0030097 × BMI2 – 0.0860806 × DBP + 0.0005269 × DBP2 + 0.0059161 × SBP + 0.0118834 × Duration of T2DM + 0.0028793 × age ×(eGFR≥60 and eGFR<90) – 0.0314914 × age ×(eGFR<60)^) × 100% |

ESRD = End Stage Renal Disease; T2DM = Type 2 Diabetes Mellitus; STDR = Sight Threatening Diabetic Retinopathy; BMI = Body Mass Index; HbA1c = Hemoglobin A1c; SBP = Systolic Blood Pressure; DBP = Diastolic Blood Pressure; ACR= Albumin/Creatinine Ratio; eGFR = estimated Glomerular Filtration Rate
